# Association of Admission Serum Calcium Levels and In-Hospital Mortality in Patients with Acute ST-Elevated Myocardial Infarction: An Eight-Year, Single-Center Study in China

**DOI:** 10.1371/journal.pone.0099895

**Published:** 2014-06-13

**Authors:** Xin Lu, Yunle Wang, Haoyu Meng, Pengsheng Chen, Yaqing Huang, Zemu Wang, Ningtian Zhou, Chunjian Li, Liansheng Wang, Enzhi Jia, Zhijian Yang

**Affiliations:** 1 Department of Cardiology, the First Affiliated Hospital of Nanjing Medical University, Nanjing, China; 2 Department of Gastroenterology, the Drum Tower Hospital of Nanjing Medical University, Nanjing, China; 3 Department of Geriatrics, The First Affiliated Hospital of Nanjing Medical University, Nanjing, China; Indiana University School of Medicine, United States of America

## Abstract

**Objective:**

The relationship between admission serum calcium levels and in-hospital mortality in patients with acute ST-segment elevation myocardial infarction (STEMI) has not been well definitively explored. The objective was to assess the predictive value of serum calcium levels on in-hospital mortality in STEMI patients.

**Methods:**

From 2003 to 2010, 1431 consecutive STEMI patients admitted to the First Affiliated Hospital of Nanjing Medical University were enrolled in the present study. Patients were stratified according to quartiles of serum calcium from the blood samples collected in the emergency room after admission. Between the aforementioned groups,the baseline characteristics, in-hospital management, and in-hospital mortality were analyzed. The association of serum calcium level with in-hospital mortality was calculated by a multivariable Cox regression analysis.

**Results:**

Among 1431 included patients, 79% were male and the median age was 65 years (range, 55–74). Patients in the lower quartiles of serum calcium, as compared to the upper quartiles of serum calcium, were older, had more cardiovascular risk factors, lower rate of emergency revascularization,and higher in-hospital mortality. According to univariate Cox proportional analysis, patients with lower serum calcium level (hazard ratio 0.267, 95% confidence interval 0.164–0.433, p<0.001) was associated with higher in-hospital mortality. The result of multivariable Cox proportional hazard regression analyses showed that the Killip's class≥3 (HR = 2.192, p = 0.026), aspartate aminotransferase (HR = 1.001, p<0.001), neutrophil count (HR = 1.123, p<0.001), serum calcium level (HR = 0.255, p = 0.001), and emergency revascularization (HR = 0.122, p<0.001) were significantly and independently associated with in-hospital mortality in STEMI patients.

**Conclusions:**

Serum calcium was an independent predictor for in-hospital mortality in patients with STEMI. This widely available serum biochemical index may be incorporated into the current established risk stratification model of STEMI patients. Further studies are required to determine the actual mechanism and whether patients with hypocalcaemia could benefit from calcium supplement.

## Introduction

Calcium, one of the most important cations, plays a critical role in cardiac contraction, enzymatic activity, and electrophysiological characteristics. The steady state of calcium flux balance is significantly necessary for myocardium [1]. Previous studies have reported that high serum calcium concentration is an independent predictor for the incidence of coronary heart disease (CHD) including acute myocardial infarction (AMI); it is also tightly tied to the cardiovascular risk factors such as hypertension, hyperglycemia, and hyperlipidemia [2]–[5]. Meanwhile, some other prior studies have demonstrated that acute hypocalcaemia is a common electrolyte disturbance of critically ill patients, particularly in patients with sepsis, acute necrotic pancreatitis, trauma, severe burns, rhabdomyolysis, as well as the systemic inflammatory response syndrome (SIRS), and it has been shown to predict increased mortality and poor outcomes [6]–[12]. As one of common urgent critically illnesses, acute ST-segment elevation myocardial infarction (STEMI) has heightened neurohormonal activation, impaired gastrointestinal function, renal insufficiency, which all could affect calcium homeostasis. However, to date, few studies are concentrated on the prognostic role of varying calcium levels in in-hospital patients with STEMI. Thus, the current evidence for an association between them remains unclear. Therefore, we performed the present analysis to evaluate the impact of the baseline serum calcium levels on the risk of in-hospital all-cause mortality for patients hospitalized with STEMI.

## Materials and Methods

### Study Population

From January 2003 to December 2010, a total of 1431 consecutive STEMI patients admitted to the First Affiliated Hospital of Nanjing Medical University were enrolled in the study. The STEMI was defined as: typical continuous chest pain>30 min with ST-segment elevation>2.0 mm in at least 2 contiguous electrocardiographic leads, and more than a two-fold elevation in the creatine kinase-MB (CK-MB) level [13]. Exclusion criteria were, presence of chest pain>24 hours, hepatic dysfunction and/or renal dysfunction, parathyroid diseases, evidence of infection within the last 2 week, history of malignancy within the past 3 years, major trauma or surgery within a week before admission and missing laboratory values. The study protocol was approved by the Ethics Committee of the First Affiliated Hospital of Nanjing Medical University (Nanjing, China). Written informed consent was received from all patients. Data obtained from medical records, laboratory investigations and clinical case histories were retrospectively reviewed. Follow-up data were collected at discharge or demise time. Among these patients, there were 1131 men and 300 women and the median age was 65 years (range, 55–74). For each patient, a routine initial clinical assessment including clinical history, physical examination, pulse oximetry, the standard 12-lead electrocardiogram (ECG) and continuous ECG monitoring, was carried out. The thrombolysis in myocardial infarction (TIMI) risk score for STEMI [14] was calculated for each subject on admission and used to assess risk within this study population.

### Blood Sampling and Laboratory Analyzes

The blood samples were collected in the emergency room from each patient after admission. In all cases, peripheral venous blood samples for hematologic and biochemical measurements were drawn. The concentrations of calcium (mmol/L), sodium (mmol/L), potassium (mmol/L), and chloride (mmol/L) were measured with the VITROS 5–1 FS chemistry system (Ortho Clinical Diagnostics, Raritan, NJ). Hypocalcaemia was defined as the concentrations of serum calcium<2.15 mmol/L according to reference range. Common blood counting parameters, in general, including the total white blood cell count, neutrophil count, eosinophil count, monocyte count, lymphocyte count, and addicted Alkaline granulocyte count, were measured by an automatic hematology analyzer (Bayer Diagnostics ADVIA120). Meanwhile, the levels of albumin (g/L), HDL-cholesterol (mmol/L), LDL-cholesterol (mmol/L) and aspartate aminotransferase (U/L) were analyzed by the AU2700 automatic biochemical analyzer.

### Statistical Analysis

Statistical analysis was performed by SPSS 16.0 (SPSS, Chicago, Illinois, USA) and SAS version 9.1 (SAS Institute Inc., Cary, NC). The Kolmogorov–Smirnov test was used to test continuous variables for normal distribution. Normally distributed data, including neutrophil count, TIMI risk score for STEMI, albumin, serum potassium, onset-arrival time and the length of hospitalization were expressed as mean ± standard deviation and comparisons were analyzed by one-way ANOVA among the quartiles. Skewed data, including age, heart rate, high density lipoprotein (HDL)-cholesterol, Low density lipoprotein (LDL)-cholesterol, Peak creatine kinase (CK), Troponin-T, left ventricular ejection fraction (LVEF), aspartate aminotransferase (AST), serum chlorine, serum sodium, were expressed as the median (inter quartile range) and compared by the Kruskal-Wallis H test. Categorical variables were summarized as percentages and compared among the groups by Chi-squared analysis. The cumulative survival curves for in-hospital mortality with different quartiles of serum calcium were constructed using the Kaplan–Meier method, and curves were compared by the log–rank test. The multivariable Cox proportional hazards regression analyses using forward likelihood ratio tests were performed to explore the independent importance of the variables for in-hospital mortality. The assumptions of proportional hazards were assessed by including time dependent covariates in the models and no indication of a violation was found. The linearity of the continuous variables was checked with Martingale residuals. To avoid over fitting, the following variables known to affect prognosis after STEMI were considered in the multivariable Cox regression analyses model: age, gender, Killip's class, LVEF, AST, neutrophil count, albumin, serum calcium level, emergency revascularization. The multivariable analyses were performed in 2 separate multivariable Cox models, with serum calcium entered as continuous data, or stratified by quartiles. All P-values were 2-tailed and P-value<0.05 was considered statistically significant.

## Results

### Baseline Characteristics

**Figure 1:**
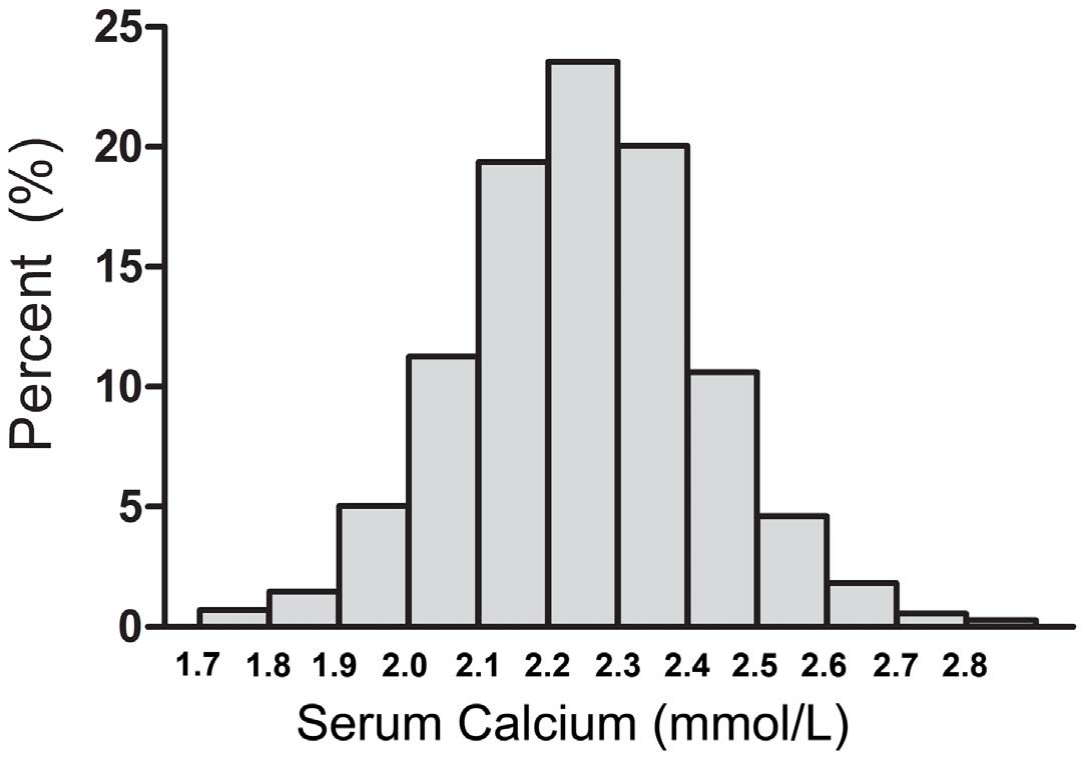
Distribution of baseline serum calcium levels at admission in 1,431 STEMI patients. The admission serum calcium levels were normally distributed with a mean admission calcium level of 2.25±0.21 (mmol/L).

In this present study, a total of 1431 patients admitted to our department with STEMI were enrolled, and the information about serum calcium of each patient was available at the time of hospital admission. The admission serum calcium levels were normally distributed (Figure 1) with a mean admission calcium level of 2.25±0.21 (mmol/L). Based on the serum calcium upon admission, patients were stratified into quartiles (1st quartile: <2.14, 2nd quartile: 2.14-<2.25, 3rd quartile: 2.25-<2.36 and 4th quartile: >2.36). The baseline characteristics of patients according to serum calcium quartiles are presented in Table 1. Median age decreased as the serum calcium increased [69(59–75), 66(57–74), 65(55–74), 60(52–70), p<0.001 for trend across quartiles], while gender showed no difference significantly across quartiles of serum calcium. The traditional risk factors of coronary artery disease including hypertension, stroke history, diabetes mellitus and smoking status, did not differ among the quartiles. Patients in the lower quartiles of serum calcium, as compared to the upper quartiles of serum calcium, were more likely to be presented with higher TIMI risk score for ST-segment MI (p<0.001) upon admission. According to laboratory data on admission, these patients were also associated with a lower level of albumin, serum sodium and serum potassium, as well as a higher level of neutrophil count and troponin-T. Moreover, the level of HDL-cholesterol, LDL-cholesterol, AST, and Killip's class≥3, differed significantly across the serum calcium quartiles (p = 0.020, p<0.001, p<0.001, p<0.001, p = 0.011, respectively). In contrast, there was no statistically significant distinction between the aforementioned groups and the infarcted region, onset-arrival times, peak CK, LVEF, as well as serum chlorine level.

**Table 1:** Comparisons of the baseline characteristics of the STEMI patients according to serum calcium.

Variables	Serum calcium	p-value
	Quartile1	Quartile 2	Quartile 3	Quartile 4	
Serum calcium, mmol/L	<2.14	2.14-<2.25	2.25-<2.36	≥2.36	
No. of patient	347	339	367	378	
Age, year	69(59–75)	66(57–74)	65(55–74)	60(52–70)	<0.001
Males	280(80.7%)	264(77.9%)	298(81.2%)	289(76.5%)	0.333
Heart rate, beats/minute	78(68–89)	74(65–85)	75(65–86)	76(65–85)	0.129
Hypertension, %	182(52.4%)	188(55.5%)	213(58.0%)	212(56.1%)	0.509
Diabetes mellitus, %	79(22.8%)	68(20.1%)	96(26.1%)	100(26.5%)	0.147
Stroke, %	18(5.2%)	20(5.9%)	18(4.9%)	21(55.6%)	0.942
Smoker, %	163(47.0%)	141(41.6%)	171(46.6%)	168(44.4%)	0.486
TIMI risk score for STEMI	4.19±2.01	3.71±1.84	3.60±1.77	3.36±1.71	<0.001
Killip's class≥3, %	30(8.6%)	16(4.7%)	14(3.8%)	29(7.7%)	0.020
Onset-arrival time, h	8.09±6.52	8.27±6.68	8.05±6.38	7.66±6.32	0.828
Peak CK, U/l	474(148–1643)	595(108–1704)	407(95–1496)	272(70–1406)	0.456
Troponin-T, ng/ml	1.2(0.4–2.0)	0.9(0.3–2.0)	0.6(0.1–1.7)	0.5(0.1–1.5)	<0.001
LVEF, %	56.2(47.7–60.2)	57.1(51.0–61.3)	56.9(49.9–60.5)	56.9(52.1–60.4)	0.334
Infarcted region					
Anterior AMI, %	197(56.8%)	182(53.7%)	205(55.9%)	231(61.1%)	0.229
Inferior AMI, %	140(40.3%)	141(41.6%)	148(40.3%)	136(36.0%)	0.426
Lateral-wall AMI, %	12(3.5%)	13(3.8%)	18(4.9%)	11(2.9%)	0.498
Laboratory data on admission					
Neutrophil count, 10∧3/µl	7.9±4.5	7.0±3.2	6.9±3.4	6.5±3.4	<0.001
Albumin, g/L	35.7±5.1	37.8±5.1	39.1±3.9	40.9±4.8	<0.001
HDL-cholesterol, mmol/L	0.95(0.81–1.16)	1.03(0.86–1.21)	1.02(0.86–1.19)	1.05(0.89–1.25)	<0.001
LDL-cholesterol, mmol/L	2.31(1.85–2.86)	2.51(2.04–3.16)	2.59(2.02–3.10)	2.65(2.16–3.30)	<0.001
AST, u/L	99(42–247)	102(37–223)	84(33–180)	69(32–211)	0.011
Serum chlorine, mmol/L	104(101–107)	104(102–107)	104(102–107)	104(101–106)	0.242
Serum sodium, mmol/L	140(137–142)	141(138–143)	141(138–143)	141(139–144)	<0.001
Serum potassium, mmol/L	4.0±0.6	4.1±0.7	4.1±0.5	4.2±0.6	0.044

Data are expressed as mean ± standard deviation for normally distributed data, median (inter quartile range) for abnormally distributed data and percentage (%) for categorical variables. CK  =  Creatine kinase; LVEF  =  Left ventricular ejection fraction; LDL  =  Low density lipoprotein; HDL  =  High density lipoprotein; AST  =  Aspartate aminotransferase.

### In-hospital Management and Clinical Outcome

**Figure 2:**
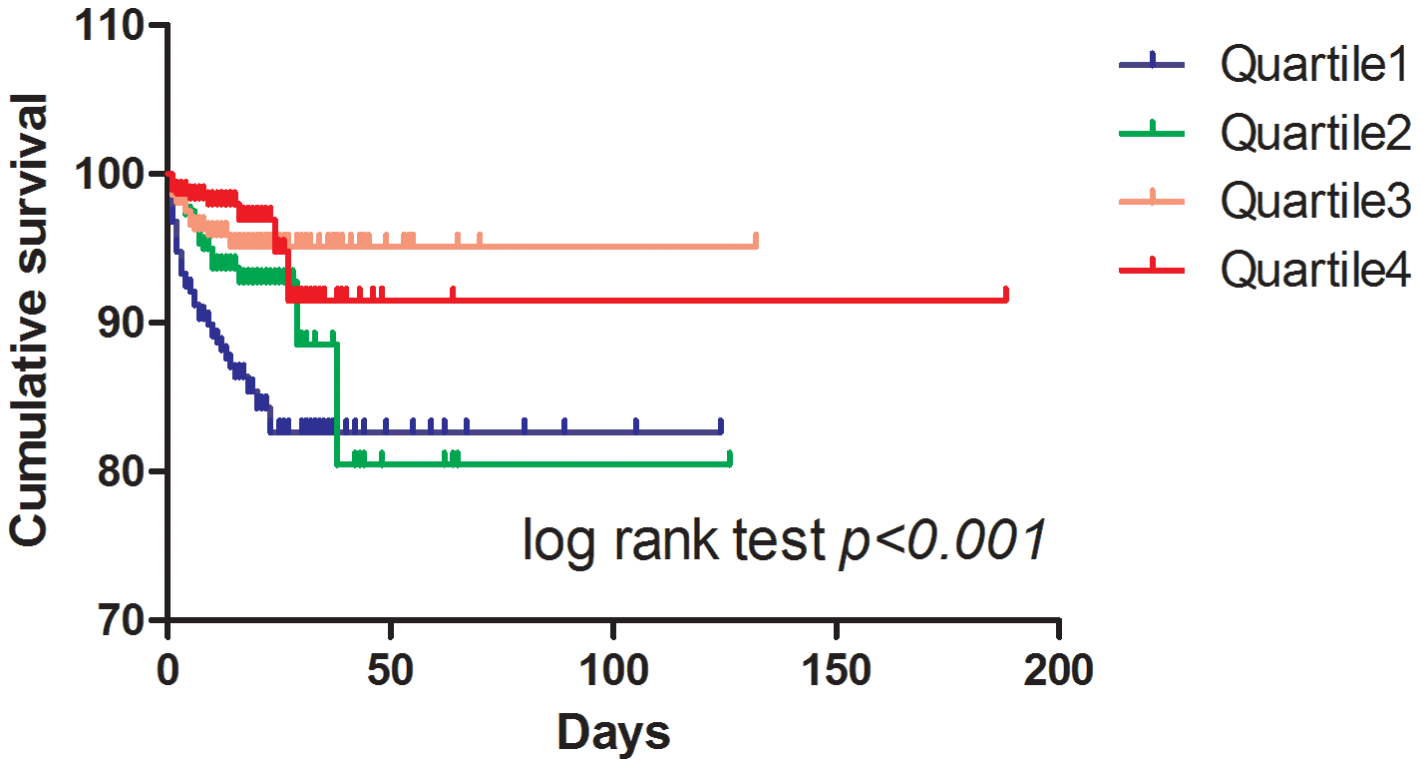
Kaplan-Meier curves for in-hospital cumulative mortality according to serum calcium of admission. Patients were stratified by quartiles of serum calcium. The comparisons among the groups were performed using the log rank test.

The in-hospital management and mortality of patients according to serum calcium quartiles are listed in Table 2. Overall, 1026 patients with STEMI underwent emergency revascularization therapy by percutaneous coronary intervention (PCI) (71.7%). The rate of emergency revascularization exhibited significant distinction among quartiles (p = 0.001 for trend across quartiles), but not for the rate of elective revascularization (p = 0.147). Furthermore, there was no significant disparity in the length of hospitalization and the administration of pharmacologic agents, including antiplatelets, beta-blockers, nitrates, angiotensin-converting enzyme inhibitors (ACEI), and/or angiotensin receptor blockers (ARB), calcium antagonists, statins, and heparin/low molecular heparin. In addition, a lower in-hospital mortality was observed as the serum calcium quartiles increased (12.7%, 6.5%, 4.4%, 2.9%, p<0.001 for trend across quartiles). Kaplan-Meier curves for in-hospital cumulative mortality showed that the mortality was higher in patients with hypocalcemia (Figure 2).

**Table 2:** In-hospital management and mortality of the STEMI patients according to serum calcium.

In-hospital Management	Serum calcium	p-value
	Quartile1	Quartile2	Quartile3	Quartile4	
Emergency revascularization	227(65.4%)	250(73.7%)	254(69.2%)	295(78.0%)	0.001
Elective revascularization	63(18.2%)	60(17.6%)	59(16.1%)	48(12.7%)	0.147
Antiplatelets	328(94.5%)	330(97.3%)	351(95.6%)	370(97.9%)	0.062
Beta-blockers	249(71.8%)	235(69.3%)	270(73.6%)	279(75.7%)	0.271
Nitrates	330(95.1%)	310(91.4%)	344(93.7%)	362(95.8%)	0.076
ACEI/ARB	256(73.4%)	264(77.8%)	290(79.0%)	299(79.1%)	0.281
Calcium antagonists	114(32.9%)	100(29.5%)	95(25.9%)	101(26.7%)	0.159
Statins	367(76.9%)	275(81.2%)	300(81.7%)	329(87.0%)	0.083
Heparin/low molecular heparin	295(85.0%)	298(87.9%)	303(82.6%)	296(88.8%)	0.056
Hospitalization (day)	15.1±13.6	14.0±11.0	13.2±11.5	13.2±12.4	0.123
In-hospital mortality (%)	44(12.7%)	22(6.5%)	16(4.4%)	11(2.9%)	<0.001

Data are expressed as mean ± standard deviation for normally distributed data and percentage (%) for categorical variables. Revascularization  =  percutaneous coronary intervention; ACEI  =  angiotensin-converting enzyme inhibitors; ARB  =  angiotensin receptor blockers.

### Survival and Predictive Factors of In-hospital Mortality

Table 3 shows univariate and multivariable Cox proportional hazard regression analyses of factors associated in-hospital mortality. For in-hospital mortality, age, gender, Killip's class≥3, neutrophil count, serum calcium, aspartate aminotransferase, albumin and emergency revascularization were analyzed using a multivariable Cox proportional hazard regression model. And then eventually, five independent predictors of in-hospital mortality emerged in this series: Killip's class≥3 [hazrad ratio (HR) 2.192, p = 0.026)], AST (HR = 1.001, p<0.001), neutrophil count (HR = 1.123, p<0.001), serum calcium level (HR = 0.255, p = 0.001), and emergency revascularization (HR = 0.122, p<0.001). In univariate analysis, every 1- µmol/L increase in serum calcium was associated with lower in-hospital mortality (HR = 0.267, 95% confidence interval (CI) 0.164–0.433, p<0.001). After adjustment for potentially confounding variables, serum calcium level was still an independent predictor of in-hospital mortality (HR = 0.255, 95% CI 0.114–0.572, p = 0.001), indicating that lower serum calcium level was associated with higher mortality probability. When stratified by quartiles, the upper quartile of serum calcium level was associated with a decreased risk of in-hospital mortality (HR = 0.753, 95% CI 0.612–0.928, p = 0.009), compared with the lowest quartile.

**Table 3:** Univariate and multivariable Cox regression analyses of factors associated in-hospital mortality.

Variables	Unadjusted HR	95% CI	p value	Adjusted HR^a^	95% CI	p-value
Age	1.059	1.040–1.078	<0.001	1.017	0.994–1.041	0.158
Gender	1.726	1.155–2.579	0.008	1.184	0.682–2.058	0.548
Hypertension	0.735	0.506–1.069	0.107			
Diabetes mellitus	1.259	0.836–1.896	0.271			
Killip's class≥3	6.169	4.092–9.300	<0.001	2.192	1.097–4.382	0.026
Peak CK	1.000	1.000–1.001	0.052			
Troponin-T	1.119	0.910–1.377	0.287			
LVEF	0.954	0.909–1.002	0.059	0.959	0.905–1.016	0.152
Anterior AMI	1.400	0.947–2.069	0.092			
Inferior AMI	0.734	0.493–1.093	0.128			
Lateral-wall AMI	0.727	0.230–2.297	0.587			
Neutrophil count	1.158	1.122–1.195	<0.001	1.123	1.076–1.172	<0.001
Platelet count	0.997	0.994–1.000	0.070			
HDL-cholesterol	0.652	0.271–1.567	0.339			
LDL-cholesterol	1.020	0.776–1.341	0.889			
Albumin	0.936	0.912–0.960	<0.001	0.994	0.948–1.041	0.787
Aspartate aminotransferase	1.001	1.001–1.002	<0.001	1.001	1.000–1.001	<0.001
Serum calcium	0.267	0.164–0.433	<0.001	0.255	0.114–0.572	0.001
Emergency revascularization	0.107	0.069–0.168	<0.001	0.122	0.068–0.217	<0.001

^a^Adjusted for age, gender, Killip's class≥3, LVEF, neutrophil count, albumin, aspartate aminotransferase, serum calcium and emergency revascularization.

## Discussion

The present study was conducted to evaluate the relationship between the admission serum calcium levels and in-hospital mortality of 1431 consecutive patients with STEMI. The result of this study documented that a decreased baseline serum calcium level measured on admission was associated with higher in-hospital all-cause mortality, even after adjusting for the possible confounding predictors. The highest mortality was observed among patients with serum calcium concentration less than 2.14 mmol/L. These findings highlighted that declined serum calcium concentration is a predictor of short term mortality for STEMI rather than just a marker of an acute medical condition. Although there was a significant higher frequency of some cardiovascular risk factors in patients with low serum calcium level, it did not interfere with the significant prognostic effect of serum calcium on in-hospital mortality among STEMI patients in the multivariable analysis.

A considerable number of clinical studies have suggested that hypocealcemia is a common electrolyte disturbance among critically ill patients and it has been shown to be associated with increased mortality [6]–[12]. Our study demonstrated, for the first time, that in STEMI patients, serum calcium held a prognostic role for in-hospital mortality. Compared with those whose serum calcium concentrations were normal, patients with hypocalcaemia tended to be older, with lower blood pressure, lower concentration of serum albumin, higher TIMI risk score for STEMI and higher neutrophil count on admission. They were also strongly associated with lower emergency revascularization rate, which could improve myocardial salvage and evidently made a difference in acute myocardial infarction (AMI) mortality [15], [16]. Numerous researchers have reported the independent predicative value of neutrophil count for in-hospital and long-term mortality in STEMI patients [17], [18] along with the mechanisms about inflammation reaction [19]. The results of our study are consistent with the above study. Moreover, it has been well established that less than half of total serum calcium is protein bound, principally to albumin [20]. In the present study, although albumin level seemed to be significantly lower in hypocalcaemia group, all data were within a small zone and the normal range. As the albumin level was also included in the multivariable Cox regression model, the interference of the albumin on serum calcium could be eliminated. In addition, another finding of this study was that AST was also a predictor for in-hospital mortality which was similar to the study by Chiara Lazzeri et al. [21].

Calcium plays a critical role in osteogenic function, signalling function [22] and enzymatic function. An increasing level of intracellular calcium in platelet is one of the most important links in atherosclerotic plaques formation or thrombogenesis process in CHD [23], thus calcium is consumed, which induces hypocalcaemia in these patients [24]. Since the emergency revascularization rate and TIMI risk score for STEMI in this study was significantly associated with serum calcium level, the assumption was that low calcium level might partially reflect worsened vascular condition in patients. The lower calcium level was, the more plaques or thrombus were formed [23], and the smaller chance of revascularization would be. In this present study, older patients had a lower level of serum calcium after STEMI, this may be associated with worse regulation of calcium, higher incidence of osteoporosis, and less chance to survive in critical ill.

The mechanism which may account for the association between the admission serum calcium levels and in-hospital mortality with STEMI was unknown. However, intracellular calcium overload may play a key role. Intracellular calcium acts as a second messenger [22] for the secretion of some hormones and neurotransmitters, as well as an intracellular permeation regulator and mediator of muscle contraction [25]. The decreased serum calcium concentration would increase the calcium channels on vascular smooth muscle cells (VSMCs), and increase the level of intracellular calcium, which is known as “the abnormal calcium influx” [26]. This change would play an important role in the process of cell migration [27] and atherosclerotic plaques formation [28]. Extracellular calcium content is nearly 10000 times over intracellular calcium and lots of complex mechanisms involved in maintaining this concentration ladder. Boya et al. reported that calcium influx would cause chondriosome swollen and then lead to a series of cellular toxin damage [29]. The dysfunction of vascular endothelial cells leads to more lipid deposition and thrombus formation [30], thus increases ionized calcium consumption. Therefore, a hypocalcaemia vicious cycle is formed. Cell toxin damage would aggravate inflammation in STEMI patients, which also plays an important role in coronary pathology and formation of plaque [31]. In this study, neutrophils count, an inflammation factor, was significantly higher in hypocalcaemia group, which was similar to Meissner et al's study [18]. Thus, the results from the above studies are consistent with those of our study where hypocalcaemia patients own worse vascular condition than those with normal calcium level, and may have more severe coronary damage and worse prognosis.

A large number of studies reached the conclusion that calcium intake were associated with the incidence of heart disease [3]–[5]. Recently, researchers claimed that both the subjects in the low and high level of serum calcium had higher rate of cardiovascular disease incidence [32]. However, the results are inconsistent between men and women. Women seem to have high ischemic heart disease (IHD) mortality in high serum ionized calcium group, while men have high risk of IHD death in low calcium group. What's more, men seem to benefit more from daily calcium supplement than women prospectively, although there were no strong evidence between dietary calcium intake and cardiovascular disease death. A reported prospective study suggested that systolic blood pressure and left ventricular stroke work index increaseds after slowly calcium intravenous injection to critically ill patients complicated by hypocalcaemia [33]. Calcium administration for STEMI patients with hypocalcaemia may be beneficial for short term outcome of post-AMI, but this still needs solid evidence to prove.

The advantages of our study are a large patient population and the comprehensive available laboratory data, but there are still some limitations in our present study. First, our study protocol is designed as a retrospective, observational, and single-center study rather than a prospective multi-center cohort study. Second, as some sufferers who died before reaching hospital, and these missing data may lead to an underestimated mortality rate in patients with STEMI. Finally, the mechanism of the associations between hypocalcaemia and in-hospital mortality in STEMI is still unclear. Although serum calcium concentration is significantly associated with in-hospital mortality in STEMI in the present study, the clinical significance of this finding needs further investigation.

## Conclusions

Serum calcium was an independent predictor for in-hospital mortality in STEMI patients. This widely available serum biochemical index may help identify high-risk STEMI individuals, who might benefit from more aggressive interventions. However, the actual pathophysiologic mechanism and whether patients with hypocalcaemia could benefit from calcium supplement requires further study.
